# When and how are complications suspected after shunt surgery in patients with normal pressure hydrocephalus?

**DOI:** 10.1007/s00701-024-06415-1

**Published:** 2025-01-08

**Authors:** Johan Virhammar, Oskar Fasth, Fredrik Vedung

**Affiliations:** 1 Department of Medical Sciences, Neurology, Uppsala University, Akademiska Sjukhuset, ing 85, Uppsala, 751 85 Sweden; 2 Department of Medical Sciences, Neurosurgery, Uppsala University, Uppsala, Sweden

## Abstract

**Purpose:**

The follow-up routine for patients with idiopathic normal pressure hydrocephalus (iNPH) after shunt surgery differs across medical centers. Shunt surgery is not without risks, with complications emerging at various times after the procedure. The aim was to explore the timing and methods of detecting complications following ventriculoperitoneal shunt surgery for iNPH.

**Methods:**

This retrospective study examined patients who underwent shunt surgery for iNPH at Uppsala University Hospital between 2011 and 2018. The cohort comprised 491 patients. Postoperative complications within the first 12 months were recorded from medical records. Complications were classified by type, and the method or event that first indicated the complication was documented.

**Results:**

Of the 491 patients, 102 (20.8%) experienced complications during the one-year follow-up period, with a shunt revision rate of 15.5% (76 patients requiring reoperation). Subdural hematomas/hygromas were the most common complications, with 27 cases; only three required surgical intervention. Most complications were identified through additional appointments triggered by patient-reported symptoms (31.4%), while the planned follow-up routine with CT scans and planned follow-up visits together accounted for 56% of the detections. The 3-month and 12-month follow-up visits detected similar proportions of complications (12.7% and 11.8%, respectively).

**Conclusion:**

The majority of the complications were detected at a planned visit or investigation. Given the cognitive impairments in iNPH patients and that signs of shunt dysfunction can be subtle, a structured follow-up routine is important for timely detection of complications. The findings suggest that both CT scans and planned follow-up visits are critical components of effective postoperative monitoring.

## Introduction

Idiopathic Normal Pressure Hydrocephalus (iNPH) primarily affects the elderly population, presenting a growing challenge in our aging world [2]. The most common symptoms of iNPH are gait, balance, cognition, and continence disturbances [1]. The standard treatment is the implantation of a shunt system, which alleviates symptoms in approximately 50–80% of patients [4, 8, 9]. However, while shunting provides significant relief to many patients, it is not without complications. Post-operative complications may occur at various time intervals following the procedure, creating challenges in their detection and management. Older studies reported high rates of complications in iNPH, but more recent studies have reported fewer complications and revision rates of around 13% [4].

Complications can arise immediately, weeks, or even years after the initial shunt surgery, making their identification and treatment more complex. Early detection is often important. For example, subdural hematomas (SDH), which most commonly occur weeks or months after shunt surgery due to over-drainage, can often be managed conservatively if found in time by adjusting the shunt setting. However, if left undetected, they can grow and necessitate evacuation through craniotomy.

Different medical centers have varying post-operative follow-up protocols. Some centers routinely investigate all patients on one or multiple occasions postoperatively using brain CT scans. In contrast, others only perform scans on patients with clinical suspicion of a complication. Moreover, the routines for follow-up visits differ significantly, with variations in the frequency of visits and the duration for which the operating center follows patients.

This study addresses when and how shunt complications are identified following shunt surgery for iNPH. The timing and methods to detect a complication are described.

## Materials and methods

### Patient sample

The patient sample included all individuals with iNPH who had undergone shunt surgery at Uppsala University Hospital between 2011 and 2018 and those who had received surgery at another hospital within the same time frame but were subsequently followed up at Uppsala University Hospital. No exclusion criteria were applied.

### Data collection

The study was designed as a retrospective, observational study. All data was collected from the participants’ medical records. The postoperative routine at Uppsala University Hospital includes a CT scan at 6 weeks postoperatively and follow-up visits to a specialized NPH team at 3 and 12 months postoperatively. At each study visit, patients are investigated by a neurologist or neurosurgeon, as well as a physiotherapist and occupational therapist. The same tests for assessing symptoms are performed both pre- and postoperatively to evaluate the response to shunting. Additionally, surgical scars and all skin in conjunction with the catheter are inspected, the abdomen is examined and the setting of the shunt is controlled. All complications detected during the first 12 months postoperatively (from surgery to the second follow-up visit) were included. The type of complication and how it was detected was documented. Complication events were divided into seven categories based on when the complication was first suspected: before discharge after the shunt surgery, during the routine CT scan 6 weeks postoperatively, at a planned follow-up visit (3 months respective 12 months postoperatively), during an additional doctor’s appointment due to patient-reported symptoms indicating a shunt complication, or during a control CT scan after shunt readjustment. There was also a category labeled “referring hospital,” which included complications initially suspected in a different region of Sweden; thus, information about how they were detected was unavailable. If a shunt complication was finally determined after an invasive test, such as the lumbar infusion test or during surgery, the event or follow-up when the dysfunction was first suspected was reported. Shunt complications were divided into eight categories: subdural hematoma (SDH), intracerebral hemorrhage (ICH), shunt obstruction, shunt infection, dislocated ventricular catheter, dislocated peritoneal catheter, intestinal perforation and kinking of the catheter. A lack of clinical improvement was not considered a complication unless evidence from a CT scan or lumbar infusion test indicated one. Some patients had more than one shunt complication during the one-year follow-up; in those cases, only the first complication was included in this study. If infusion tests revealed underfunction but no clear cause of the dysfunction was detected, the complications were classified as shunt obstruction. The Swedish Ethical Review Authority approved the study.

### Statistics

Descriptive statistics were used to present the data about types of shunt complications, how they were detected, and the occurrence of shunt revision due to a complication. All statistical analyses were performed using IBM SPSS Statistics (version 28.0.1.0).

## Results

The total amount of patients included in this study was 491. The median age was 75 years (range 50–89). All but two patients received a ventriculoperitoneal (VP) shunt, and only two patients were implanted with a ventriculoatrial (VA) shunt. The study group's characteristics are presented in Table 1. 464 (95 %) of the patients were investigated at a first postoperative follow-up with a median time to follow-up 3 months (IQR: 2–3) and 415 (85%) were investigated at a second follow-up with a median time to follow-up 12 months postoperatively (IQR: 12–13). The most common shunt type was Medtronic Strata valves (Strata II or Strata MR) implanted in 483 patients (98%) with initial setting 1.0 in 2 cases (0.5%), 1.5 in 411 patients (85%) and 2.0 in 70 patients (14.5%). Other valve types were Miethke ProGAV (5 patients (setting 10–12 cm H2O)), Codman Certas (2 patients (setting 2 and 3)), and Codman Hakim (1 patient (setting 12 cm H2O)).

**Table 1:** Demographic data

	All patients (*n* = 491)
Age, years, median (range)	75 (50–89)
Gender, No. of females (%)	211 (43.0%)
Gender, No. of males (%)	280 (57.0%)
BMI, kg/m^2, median (IQR)	27 (24.6–30.1)
iNPH scale score baseline, median (IQR)	49,7 (36.9–61.7)
Time to surgery, months, median (IQR)	6 (4–8)
VP shunt, n (%)	489 (99.6%)
VA shunt, n (%)	2 (0.4%)

Abbreviations: *IQR*=interquartile range; *BMI*=body mass index; *iNPH*=idiopathic normal pressure hydrocephalus; *VP*=ventriculoperitoneal; *VA*=ventriculoatrial

**Fig. 1:**
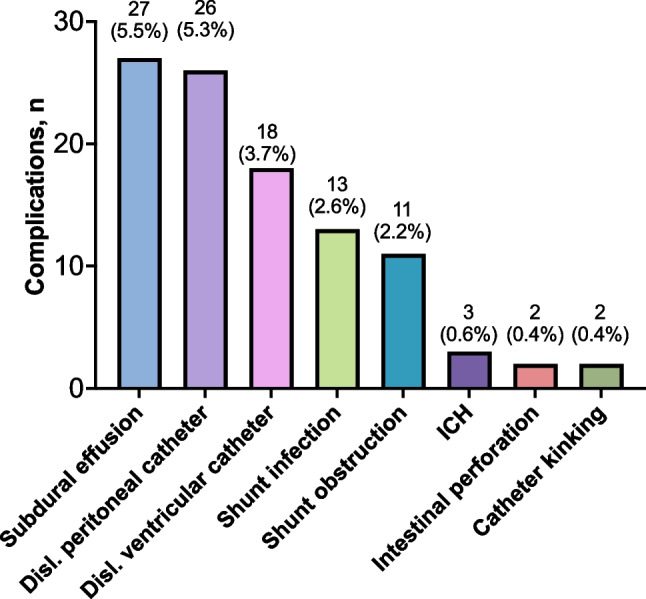
Frequency, n (%), of different shunt complications diagnosed the first 12 months after shunt surgery

**Fig. 2:**
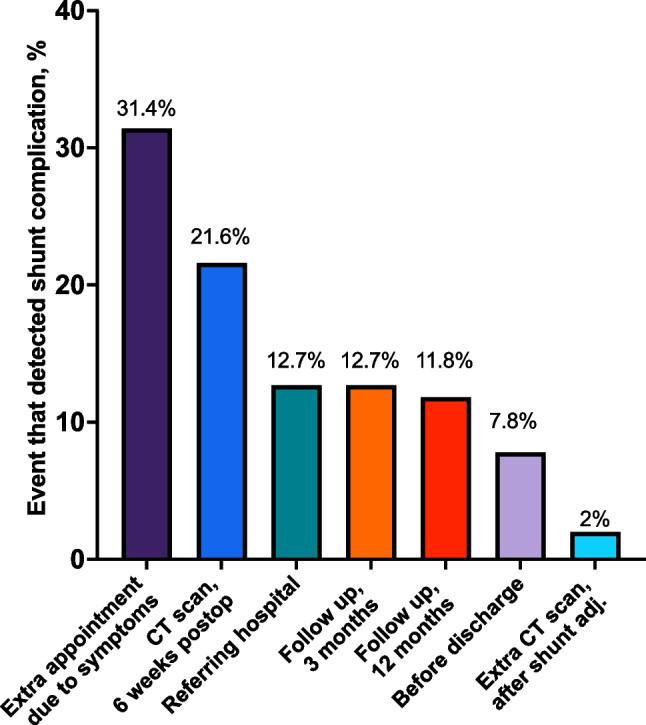
The method or event when a complication was first suspected or detected in the first 12 months after shunt surgery. The total number of complications was 102. “Referring hospital” included complications that were initially suspected in a different region of Sweden. “Before discharge” refers to complications that were detected before discharge after shunt surgery

Shunt complications were detected in 102 patients during the one-year follow-up period, making the total shunt complication rate 20.8%. Asymptomatic subdural collections were included among those complications. The shunt revision rate was 15.5%; thus, 76 of the shunt complications required reoperation. Only 3 of the 27 subdural hematomas/hygromas required evacuation of the hematoma (11%). Figure 1 illustrates the diagnosed shunt complications during the first 12 months postoperatively and Fig. 2 illustrates when or how the complications were first suspected.

## Discussion

Shunt complications within the initial postoperative year were examined in a single-center study involving 491 patients with idiopathic normal pressure hydrocephalus. The present study provides insights into the timing and methods for detecting complications. The overall shunt complication rate stood at 21%, with subdural effusions emerging as the most prevalent complication, affecting 5.5% of patients. A structured follow-up regimen, including CT scans and planned visits, facilitated the identification of 56% of the complications. In parallel, unplanned investigations initiated in response to patient-reported symptoms revealed 31% of the identified complications.

The rates of complications observed in the present study (20.8%, with 15.5% requiring revisions within 12 months) align with other modern studies and remain considerable. In a meta-analysis from 2019 by Giordan et al., the revision rate for VP-shunts in 1812 patients was 18%, with subdural effusions as the most prevalent complication, similar to our study [4]. Most complications were detected at the planned follow-up events, including clinical evaluation before discharge, postoperative CT scan, and two in-office visits. Among these, the CT scan was most important, detecting 22% of the complications. It is worth noting that both follow-up visits, 3 and 12 months postoperatively, lead to the detection of an equal proportion of complications. This indicates that the routine follow-up should not be too short, and despite one postoperative CT scan and one visit at 3 months postoperative, 12% of complications were detected at a later follow-up. However, our findings likely reflect how our follow-up is organized, and the timing of our assessments may have influenced the detection rate. Perhaps even more late complications would emerge with extended patient monitoring. Some centers also perform an early postoperative CT scan, but in one study with 68 iNPH patients, no complication was detected in the early scans [6]. This is in contrast to the 6-week CT scan performed at our center, which detected 21% of the total number of complications. Possibly, the majority of over-drainage complications are evident only after several weeks.

The most prevalent complication was subdural collections, which affected 5.5% of patients. This included both subdural hematomas and hygromas. Only 11% of the subdural collections required surgical intervention; the rest could be handled with adjustments of the valve settings.

There is no consensus on when and for how long the postoperative follow-up in patients with iNPH should be [7]. Most centers follow patients for up to one year postoperatively, but there are a few studies with longer follow-up of up to 10 years [5] and although less frequent than in the early phase, complications can be detected several years postoperatively [3].

In our cohort, a noteworthy observation is that a substantial number of complications were identified based on patient-reported symptoms, necessitating additional appointments or CT scans. It is important to acknowledge that patients with cognitive dysfunction, characteristic of iNPH, may not automatically seek healthcare for subtle symptoms. Deterioration of symptoms caused by shunt obstruction in patients with iNPH can be subtle, slow, and hard to detect. Therefore, our findings underscore the importance of a systematic follow-up routine for patients with iNPH, ensuring comprehensive detection of complications in all patients.

Some limitations should be considered. Our results are mostly relevant for centers primarily using VP-shunts since the panorama of complications can differ when using VA- or lumboperitoneal shunts. Symptomatic overdrainage without subdural effusions was not included as a complication in this study, as it would be challenging to assess retrospectively from patient records. On the other hand, all CT-verified subdural collections were categorized as complications, even though some NPH patients may experience marked improvement despite CT-verified overdrainage. Naturally, the timing of the detection of complications in this study is influenced by how the postoperative routine is designed at our center. Fewer CT-scans would have detected fewer subdural hematomas, and extended follow-up visits might have detected even more late complications. Nevertheless, the findings of our study underscore the importance of a standardized, systematic follow-up routine that includes both imaging and clinical assessments at specific intervals postoperatively. This approach is necessary for timely identification, particularly in a patient population where complications may manifest with subtle symptoms. When it comes to complications, it is evident that if you do not look for them, you will not necessarily find them. Future studies should investigate optimal time points for postoperative CT-scans and follow-up visits. 

## Data Availability

No datasets were generated or analysed during the current study.

## Abbreviations

IQR: interquartile range
BMI: body mass index
iNPH: idiopathic normal pressure hydrocephalus
VP: ventriculoperitoneal
VA: ventriculoatrial
